# Prevention of Bile Leak after Liver Surgery: A Fool-proof Method

**DOI:** 10.4103/1319-3767.48972

**Published:** 2009-04

**Authors:** Aswini K. Pujahari

**Affiliations:** Department of Surgery, Armed forces Medical College, Pune and Command Hospital (AF), Bangalore, India

**Keywords:** Bile leak, transcystic saline injection, hepatic resection

## Abstract

**Background/Aim:**

Bile leak is not uncommon after liver surgeries. There is no adequate method described to prevent this morbid complication.

**Materials and Methods:**

At the end of the liver procedure, transcystic normal saline was injected under pressure with distal clamping. Leaking saline on the cut surface of the liver was sutured. The process was repeated till no leaking was observed. A suction drain was kept for any bile leak.

**Results:**

Open liver resection and hydatid cyst surgery cases were included. There were 24 cases, with 13 males and 11 females. The age range was from 4 to 80 years, with a mean of 48 years (SD ± 17.7). The number of leak sites that could be sutured were 0-4 (mean of 2.3 ± 0.5). None had bile leak postoperatively.

**Conclusion:**

Transcystic injection under pressure with distal clamping demonstrates the leak sites. Suturing them prevents the postoperative bile leak.

Bile leak is not uncommon after hepatic surgery. The occurrence varies from 3.9[1] to 24%.[2] Leak takes place from the resected surface.[3] Sphincterotomy with endoprosthesis or endoprosthesis alone is equally effective in the management of postoperative bile leak due to various liver surgeries and injuries.[4–7] Percutaneous drainage can be added for any intra-abdominal or intrahepatic collection.[8] Prevention of bile leak was attempted by retrograde transhepatic biliary drainage (RTBD) after choledochotomy, but the leak rate was 21%, which was not different from the non-RTBD group.[9] So far, no fool-proof method of prevention of bile leak has been described.

## MATERIALS AND METHODS

**Figure 1:**
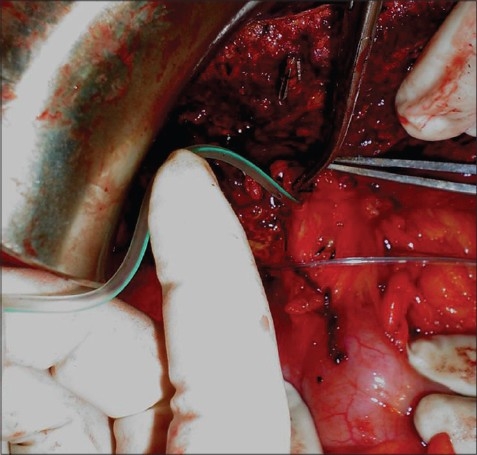
Intraoperative preparation for the bile leak test

Cases of open liver surgery from May 2000 to November 2007, involving resection and hydatid cyst, were included in the prospective study [Table 1]. Rooftop incision with upward midline extension (Mercedes Benz) was used in all liver resection cases. Mobilization of the liver was performed by cutting the triangular and falciform ligament up to the suprahepatic cava. Cholecystectomy was an initial part of all major hepatic resections. The cystic duct stump was kept long. Inflow and outflow vascular clamping were performed on the resection side only in major resections and no clamping was performed on the nonresectional side of liver. A majority of the procedure was carried out by the clamp crushing method. After June 2006, the harmonic scalpel was used (three cases) at the peripheral part of the liver and the clamp crushing method was used at the central part of the liver where larger vascular and biliary channels were seen. ***En mass*** pedicle ligation was performed along the line of resection and then the inflow clamp was removed. The cystic duct was dilated till the entry to the common duct for easy canulation of the 12 F infant feeding tube [Figure 1]. The cystic duct was ligated over the infant feeding tube. The distal part of the duct was clamped ***en mass*** along with the portal vein intermittently with a Satinsky vascular clamp 1–2 min each time during the saline injection of 3–5 mL, under pressure. This exposes the open bile ducts on the cut surface of the liver in liver resection and in the cyst cavity in hydatid cystectomy in the form of a saline leak, which is then sutured with 3-0 silk in a figure of eight manner. During the application of the suture the clamp was taken off. This process was repeated till all the leakage stopped. When no leak was observed, the tube was withdrawn, the cystic duct was ligated and the wound closed with a suction drain close to the cut surface of the liver. All patients received the usual postoperative care. The drain fluid was noted for any bile leak.

**Table 1:** Cases disposition - hepatic surgery

	RT	LT	Segmental resection	Non resection
Hepatocellular carcinoma	5	1	X	X
Cholangiocarcinoma (peripheral)	1	X	X	X
Haemangioma	X	3	X	X
Polycystic liver disease (Rt lobe only)	1	X	X	X
Hydatid cyst	1	1	4	4
Carcinoma gall bladder	1	X	X	X
Mesenchymal hamartoma	X	X	X	X
Tuberculosis*	-	-	2	-
Total	9	5	6	4

n=24

* CT reported as CaGB

## RESULTS

There was 24 cases with a age range of 4–80 years (mean 48 years, SD *±* 17.7). There were 13 males and 11 females. Breakdown of surgeries is described in Table 1. Saline leak was seen in 23 of the 24 cases. Leakage was mostly around the hilum and at the mid zone of the liver stump. The number of leak sites was 0–4 (mean of 2.3 *±* 0.5). All the leak sites required more than one stitch. Postoperatively, none had bile leak and the drain was removed after 72 h.

## DISCUSSION

Bile leak remains a serious complication. After hepatectomy, it adds to the morbidity and prolongs hospitalization[1] of the patient. Occurrence of bile leak is minimal in hepatic surgery performed using new technology like ligasure, with an occurrence varying from 0[10] to 3%.[11] Liver resection even with the use of a stapler had a bile leak of 8%.[12] No postoperative bile leak was seen after pretransaction radiofrequency coagulation.[13] It was reported to be 30% after hydatid liver surgery.[14] Saline jet was seen clearly in the smooth cyst lumen in this study. The mainstay for management of bile leak in liver resection is endoscopic retrograde cholangio pancreaticogram (ERCP) with common bile duct (CBD) stenting after pappilotomy.[4–7,15] Various other methods described are acetic acid ablation[16] and histacryl glue embolization.[17] Whenever there is a doubt of bile leak, a drain should be used. When no drain was used, the abdominal complication was 6.7%.[18] When the drain is kept longer even after ERCP and CBD stenting, the mortality is decreased.[3] In this study, the drain was kept for 72 h as no one had bile leak. Only one study addresses prevention of bile leak after liver resection by using a RTBD via a tube inserted through a choledochotomy, where the bile leak was not different from the non-RTBD group. But the bile leak was of a significantly shorter duration in the RTBD when compared with the tubeless group, i.e. 13.3 days vs 51.3 days, with few patients developing peritonitis after removal of the tube.[9] This study had the disadvantage of performing choledochotomy and keeping a transhepatic tube,[9] which offers almost the same resistance as the sphincter of oddi, because of which the leak persists. Our study was much simpler without any intrahepatic/intraductal tube. Distal clamping of the bile duct served two purposes: that of adding resistance and not allowing the tube to go beyond. Only one study involved a bile leak test via the cystic duct and reported a 41% bile leak in the test group without finding any significant advantages, with the median leak site being only one,[19] whereas we had a median of 2.29 and most of the perihilar leak requiring two sutures per leak site for complete stoppage. We used a distal clamp so that the intrabiliary pressure rose above that of the sphincter during the injection of saline so as to withstand the physiologically active sphincter of oddi. The disadvantages of this study include the need for cholecystectomy and not having measured the saline pressure, since the use of a pressure device during surgery posed technical problems. But it preserved the sphincter and avoided uncomfortable postoperative ERCP.

## CONCLUSION

Transcystic saline injection under pressure with distal clamping demonstrates the leak sites. Proper suturing prevents the postoperative bile leak after liver surgery.

## References

[1] Frena A, Martin F (2006). How to improve bilio-stasis in liver surgery. Chir Ital.

[2] Kim J, Ahmad SA, Lowy AM, Buell JF, Pennington LJ, Soldano DA (2003). Increased biliary fistulas after liver resection with the harmonic scalpel. Am Surg.

[3] Reed DN, Vitale GC, Wrightson WR, Edwards M, McMasters K (2003). Decreasing mortality of bile leaks after elective hepatic surgery. Am J Surg.

[4] Agarwal N, Sharma BC, Garg S, Kumar R, Sarin SK (2006). Endoscopic management of postoperative bile leaks. Hepatobiliary Pancreat Dis Int.

[5] Llach J, Bordas JM, Elizalde JI, Enrico C, Ginès A, Pellisé M (2002). Sphincterotomy in the treatment of biliary leakage. Hepatogastroenterology.

[6] Bridges A, Wilcox CM, Varadarajulu S (2007). Endoscopic management of traumatic bile leaks. Gastrointest Endosc.

[7] Katsinelos P, Kountouras J, Paroutoglou G, Beltsis A, Zavos C, Chatzimavroudis G (2006). The role of endoscopic treatment in postoperative bile leaks. Hepatogastroenterology.

[8] Lubezky N, Konikoff FM, Rosin D, Carmon E, Kluger Y, Ben-Haim M (2006). Endoscopic sphincterotomy and temporary internal stenting for bile leaks following complex hepatic trauma. Br J Surg.

[9] Nakai T, Kawabe T, Shiraishi O, Shiozaki H (2004). Prevention of bile leak after major hepatectomy. Hepatogastroenterology.

[10] Romano F, Franciosi C, Caprotti R, Uggeri F, Uggeri F (2005). Hepatic surgery using the Ligasure vessel sealing system. World J Surg.

[11] Saiura A, Yamamoto J, Koga R, Sakamoto Y, Kokudo N, Seki M (2006). Usefulness of LigaSure for liver resection: Analysis by randomized clinical trial. Am J Surg.

[12] Schemmer P, Friess H, Hinz U, Mehrabi A, Kraus TW, Z'graggen K (2006). Stapler hepatectomy is a safe dissection technique: analysis of 300 patients. World J Surg.

[13] Sturgeon C, Helton WS, Lamba A, Chejfec G, Espat NJ (2003). Early experience employing a linear hepatic parenchyma coagulation device. J Hepatobiliary Pancreat Surg.

[14] Uhl W, Löffler H, Zimmermann A, Tcholakov O, Gloor B, Büchler MW (1999). Surgical treatment of echinococcosis of the liver. Swiss Surg.

[15] Bhattacharjya S, Puleston J, Davidson BR, Dooley JS (2003). Outcome of early endoscopic biliary drainage in the management of bile leaks after hepatic resection. Gastrointest Endosc.

[16] Park JH, Oh JH, Yoon Y, Hong SH, Park SJ (2005). Acetic acid sclerotherapy for treatment of a biliary leak from an isolated bile duct after hepatic surgery. J Vasc Interv Radiol.

[17] Kiltz U, Baier J, Adamek RJ (1999). Selective embolization of a bile leak after operative resection of an echinococcal cyst. Dtsch Med Wochenschr.

[18] Franco D, Karaa A, Meakins JL, Borgonovo G, Smadja C, Grange D (1989). Hepatectomy without abdominal drainage: Results of a prospective study in 61 patient. Ann Surg.

[19] Ijichi M, Takayama T, Toyoda H, Sano K, Kubota K, Makuuchi M (2000). Randomized trial of the usefulness of a bile leakage test during hepatic resection. Arch Surg.

